# Preoperative rectus femoris muscle ultrasound, its relationship with frailty scores, and the ability to predict recovery after cardiac surgery: a prospective cohort study

**DOI:** 10.1186/s13741-024-00401-y

**Published:** 2024-05-23

**Authors:** Derek King Wai Yau, James Francis Griffith, Malcolm John Underwood, Gavin Matthew Joynt, Anna Lee

**Affiliations:** 1 Department of Anaesthesia and Intensive Care, The Chinese University of Hong Kong, 4/F Main Clinical Block and Trauma Centre, Prince of Wales Hospital, Shatin, NT Hong Kong SAR; 2 Department of Imaging and Interventional Radiology, The Chinese University of Hong Kong, G/F Prince of Wales Hospital, Shatin, NT Hong Kong SAR; 3 Cardiovascular Services, Auckland City Hospital, Auckland District Health Board, Auckland, New Zealand

**Keywords:** Muscle ultrasonography, Frailty, Cardiac surgery, Diagnostic accuracy

## Abstract

**Background:**

Frailty is common in patients undergoing cardiac surgery and is associated with poorer postoperative outcomes. Ultrasound examination of skeletal muscle morphology may serve as an objective assessment tool as lean muscle mass reduction is a key feature of frailty.

**Methods:**

This study investigated the association of ultrasound-derived muscle thickness, cross-sectional area, and echogenicity of the rectus femoris muscle (RFM) with preoperative frailty and predicted subsequent poor recovery after surgery. Eighty-five patients received preoperative RFM ultrasound examination and frailty-related assessments: Clinical Frailty Scale (CFS) and 5-m gait speed test (GST_5m_). Association of each ultrasound measurement with frailty assessments was examined. Area under receiver-operating characteristic curve (AUROC) was used to assess the discriminative ability of each ultrasound measurement to predict days at home within 30 days of surgery (DAH_30_).

**Results:**

By CFS and GST_5m_ criteria, 13% and 34% respectively of participants were frail. RFM cross-sectional area alone demonstrated moderate predictive association for frailty by CFS criterion (AUROC: 0.76, 95% CI: 0.66–0.85). Specificity improved to 98.7% (95% CI: 93.6%-100.0%) by utilising RFM cross-sectional area as an ‘add-on’ test to a positive gait speed test, and thus a combined muscle size and function test demonstrated higher predictive performance (positive likelihood ratio: 40.4, 95% CI: 5.3–304.3) for frailty by CFS criterion than either test alone (*p* < 0.001). The combined ‘add-on’ test predictive performance for DAH_30_ (AUROC: 0.90, 95% CI: 0.81–0.95) may also be superior to either CFS or gait speed test alone.

**Conclusions:**

Preoperative RFM ultrasound examination, especially when integrated with the gait speed test, may be useful to identify patients at high risk of frailty and those with poor outcomes after cardiac surgery.

**Trial registration:**

The study was registered on the Chinese Clinical Trials Registry (ChiCTR2000031098) on 22 March 2020.

**Supplementary Information:**

The online version contains supplementary material available at 10.1186/s13741-024-00401-y.

## Background

Frailty is common in patients undergoing cardiac surgery, with a prevalence of 20% to 50% (Shimura et al. 2017; Afilalo et al. 2012; Sündermann et al. 2011). However, frailty is often underdiagnosed as preoperative risk stratification tools are complex and may fail to accurately and consistently identify frailty (Koh and Hwang 2019; Bissot et al. 2016). In addition, no consensus exists regarding the optimal diagnostic criteria for frailty (Aucoin et al. 2020; Dent et al. 2016; Rodríguez-Mañas et al. 2013). Subjective and objective assessment tools, such as the Clinical Frailty Scale (CFS) (Rockwood et al. 2005) and the 5-m gait speed test (GST_5m_) (Afilalo et al. 2010) respectively, are widely used alone or in multicomponent frailty criteria (Koh and Hwang 2019). As frail patients are at higher risk of an adverse postoperative outcome, prolonged hospital stay, increased short-term and long-term mortality, and higher healthcare resource utilisation (Shimura et al. 2017; Aucoin et al. 2020; Goldfarb et al. 2017; Kim et al. 2016), timely identification of such patients who could potentially benefit from prehabilitation programs is important (Yau et al. 2019).

Sarcopenia, the process of degenerative change in muscle mass and density associated with reduced muscle strength or physical performance, is a major contributor to frailty (Cruz-Jentoft et al. 2019; Landi et al. 2015). Skeletal muscle mass assessment is an objective criterion which could potentially serve as an improved, or alternative marker to current frailty scoring systems. Although computerised tomography and magnetic resonance imaging are the gold standards for muscle morphology assessment, these techniques are not routinely used in clinical practice owing to high technical complexity, cost, and lack of portability (Cruz-Jentoft et al. 2019). In contrast, ultrasound with its potential ability to assess macroscopic structural changes in skeletal muscle, is easily accessible, non-invasive, radiation-free, and relatively inexpensive. Tested against the CFS as the gold standard for frailty, ultrasound-derived measures of rectus femoris and quadriceps muscles were recently shown to have promising discriminative performances (area under receiver-operating characteristic curve [AUROC] of 0.70 and 0.80 respectively) in a mixed preoperative cohort (Canales et al. 2022). More importantly, the combination of an ultrasound-derived measure used as an add-on test (Hayen et al. 2010) to other objective frailty tests may result in a potentially higher overall diagnostic accuracy performance than using either index test alone.

The primary objective of this study was to evaluate the relationship between three ultrasound-derived measures of the rectus femoris muscle (RFM), namely muscle thickness (MT_RFM_), cross-sectional area (CSA_RFM_) and echogenicity (Echo_RFM_), and existing frailty assessment tools (CFS and GST_5m_) in patients awaiting cardiac surgery. The secondary objective was to assess the predictive performance of the ultrasound measurements for predicting patient-centred postoperative recovery up to 30 days after surgery, compared with, or in addition to the two index tests, CFS and GST_5m_.

## Methods

### Study design and participants

This was a prospective cohort study of 85 adults undergoing elective cardiac surgery at a university teaching hospital between April 2020 and May 2021. The study was reported according to the STROBE guidelines (Elm et al. 2008) and registered on the Chinese Clinical Trials Registry (ChiCTR2000031098). Approval for the study was obtained from the Joint Chinese University of Hong Kong – New Territories East Cluster Clinical Research Ethics Committee (CRE no.: 2019.711). All adult patients scheduled for elective cardiac surgery gave written informed consent for the study.

Patients were admitted to the cardiothoracic surgical ward a day before surgery and were admitted to the intensive care unit (ICU) for early postoperative care with later care in a high dependency cardiac ward. Patients who were undergoing elective coronary artery bypass grafting, valve surgery or aortic intervention were included. Patients undergoing emergency cardiac surgery; patients with known musculoskeletal or neurological disorders that were associated with lower limb muscle atrophy (e.g. poliomyelitis, stroke, peripheral neuropathy), or previous major surgery of a lower extremity (e.g. hip replacement, metal fixation, amputation), localised infection, skin disorders, and cognitive impairment (inability to provide consent) were excluded.

### Standardised ultrasound examination

Standardised ultrasound examination was performed on all recruited patients one to ten days before surgery. Ultrasound measurements of the RFM (MT_RFM_, CSA_RFM_ and Echo_RFM_) were performed by a physiotherapist who had certain previous experience of soft tissues ultrasound assessment. Under the guidance of a certified specialist radiologist, the study ultrasound operator was instructed in hands-on ultrasound of the RFM for three sessions (each lasting 60 min) with three patients scanned for this learning exercise. Subsequently, five patient scans were performed under supervision before independent scanning proceeded.

**Fig. 1:**
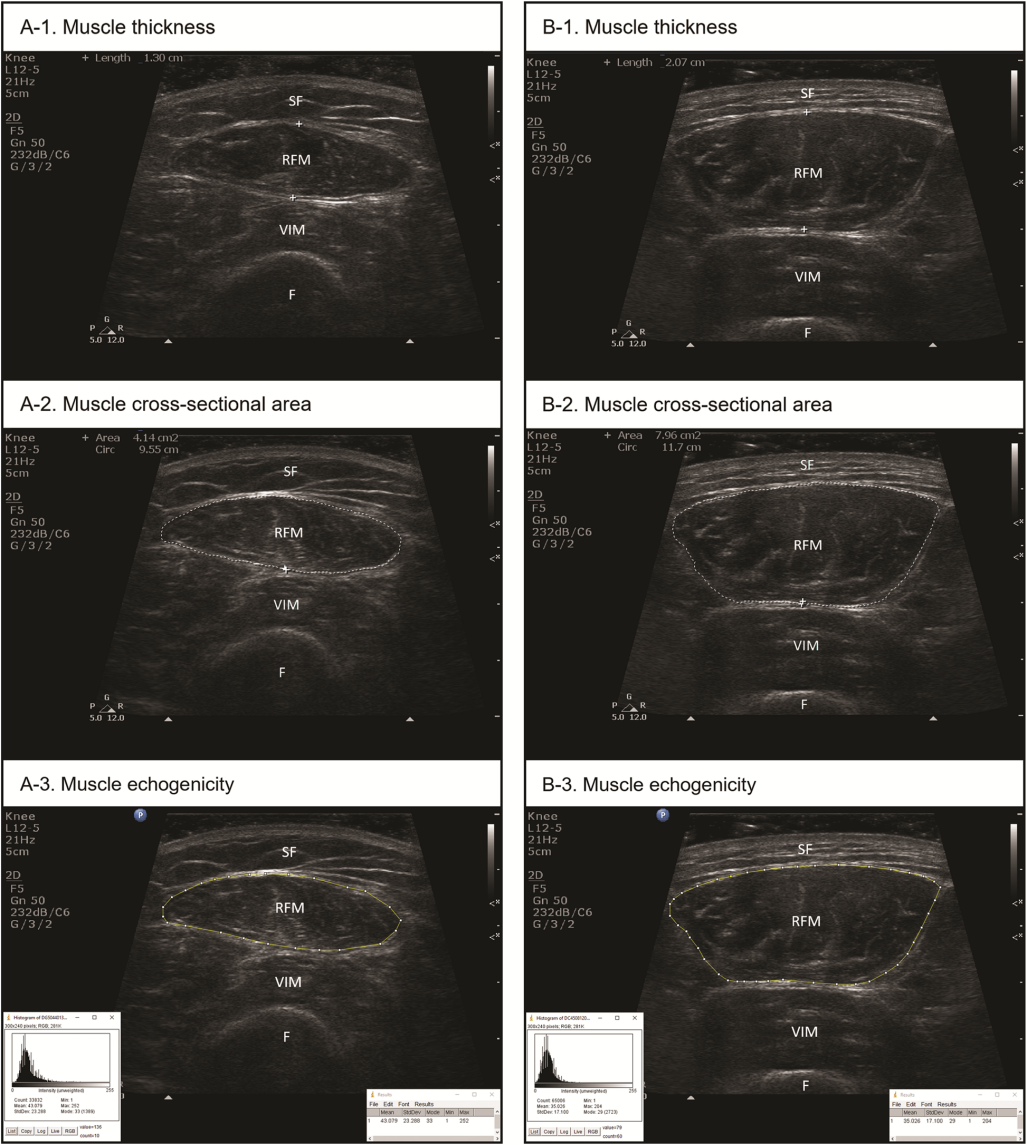
Typical transverse ultrasound images of (A) frail and (B) non-frail participants. (A-1) muscle thickness, (A-2) cross-sectional area and (A-3) echogenicity of rectus femoris muscle of a 62-year-old frail male participant. (B-1) muscle thickness, (B-2) cross-sectional area and (B-3) echogenicity of rectus femoris muscle of a 61-year-old non-frail male participant. F, femur; RFM, rectus femoris muscle; SF, subcutaneous fat; VIM, vastus intermedius muscle

**Fig. 2:**
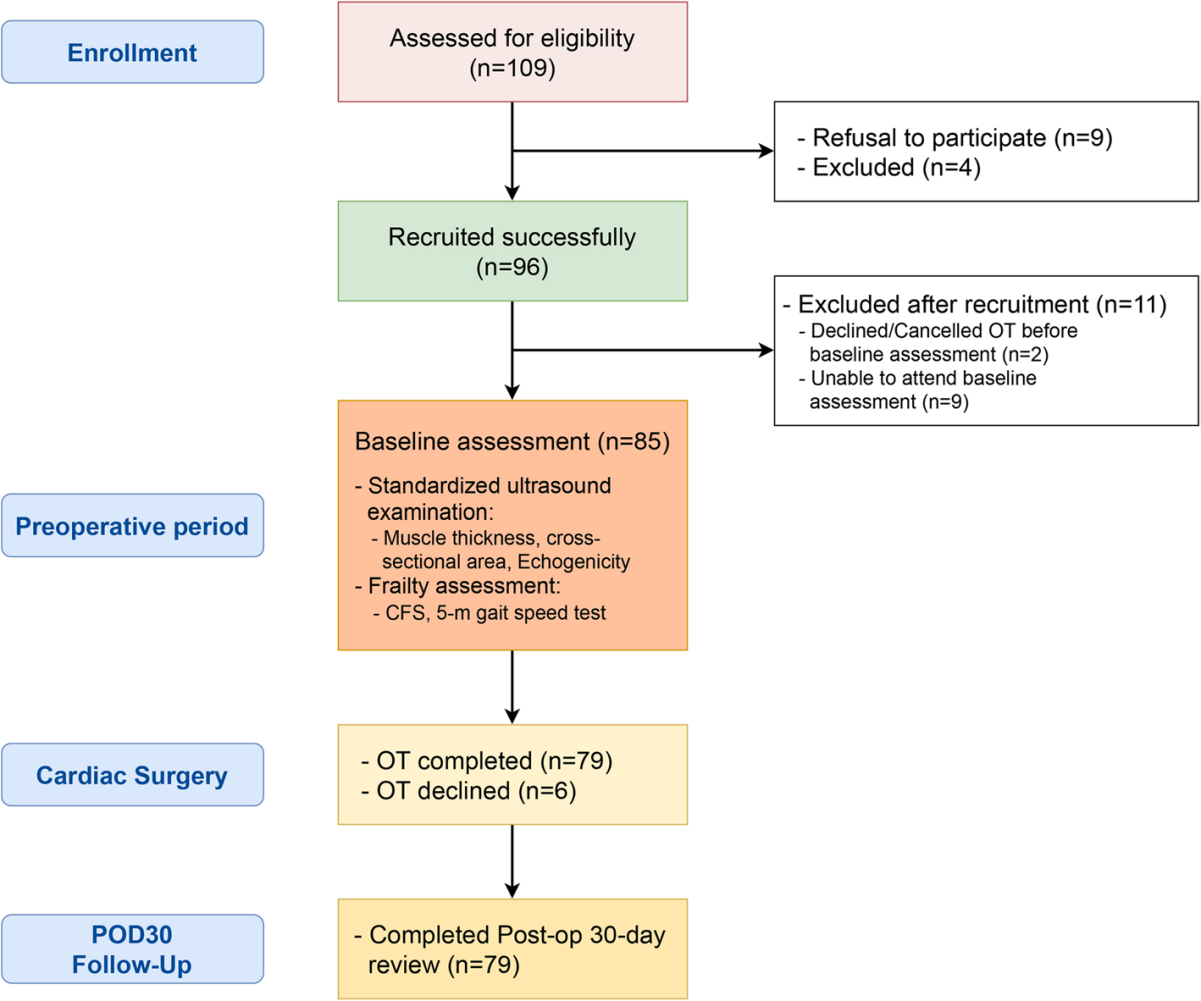
Study flow diagram

Each participating patient underwent RFM measurements on the enrolment day. The ultrasound technique utilised the B-mode of the HD11 XE ultrasound system (Philips Healthcare, Best) and a linear multi-frequent transducer (5–12 MHz, Philips Healthcare, Best). Participants were positioned lying supine in a relaxed position with both knees supported with a rolled towel in extension (in the natural resting position of 15 degrees) and the toes pointing upwards. Measurements were taken at the halfway point between the greater trochanter of femur and the proximal border of the patella (Perkisas et al. 2021). The transducer was placed perpendicular to the long axis of thigh with ample use of transmission gel to maintain acoustic contact with the skin surface and applying minimal pressure on the thigh soft tissues. The mid-portion of the RFM myofascia was used as the boundary for muscle thickness (Fig. 1A-1 and B-1) and cross-sectional area measurements (Fig. 1A-2 and B-2). Three consecutive measurements were obtained on each leg and the mean MT_RFM_ and CSA_RFM_ of both legs combined were used. To address inherent phenotypic variation in muscle mass across different body physiques, measurements of MT_RFM_ and CSA_RFM_ were normalised by adjusting for body mass index (BMI) and body surface area (BSA) with normalised values reported for analysis and comparison.

**Fig. 3:**
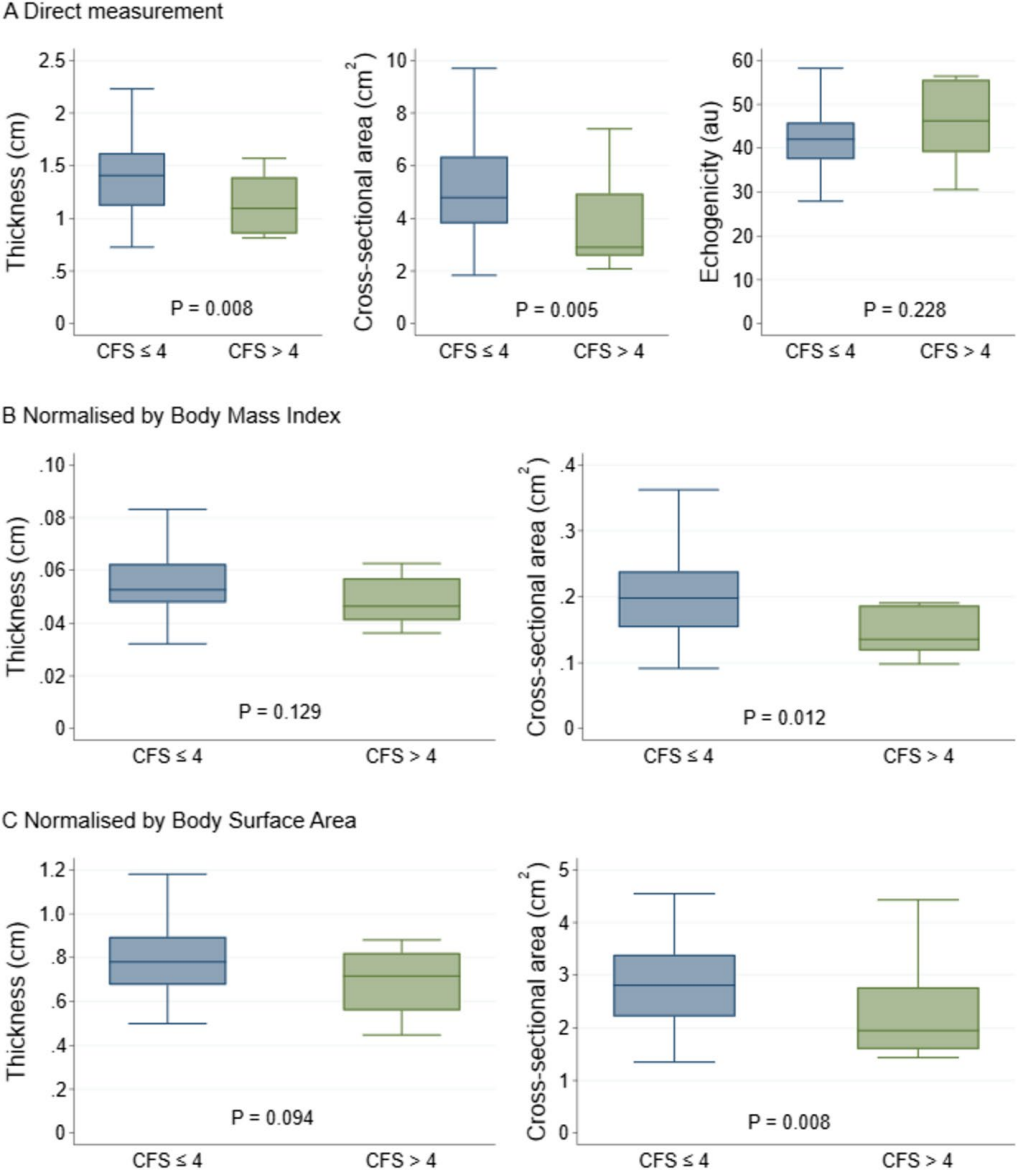
Box-and-whisker plots showing differences in ultrasound measurements between frail (CFS > 4) and non-frail (CFS ≤ 4) participants. **A** Actual values from direct measurement **B** normalised values by body mass index **C** normalised values by body surface area

Depth, overall gain, and time-gain compensation settings were kept constant when capturing images for echogenicity measurement. Images were processed with image normalisation, which is an image processing technique that distributes image intensities evenly by setting the maximum and minimum intensity in the image as 0–255 arbitrary units [au] (with background, black = 0 au and text, pure white = 255 au respectively) (Li et al. 2015; Li et al. 2012), before intensity measurement using ImageJ software version 1.52 (National Institute of Health, Bethesda). Echogenicity, i.e. the mean pixel intensity within a given region of interest, was calculated using histogram analysis and expressed in grayscale values from 0 to 255 au. In each image, a ‘Polygon selection’ tool was used to outline a region of interest within the confines of the RFM myofascia (Fig. 1A-3 and B-3). The average value of three echogenicity measurements was used.

### Outcome measures

Preoperative frailty status was assessed before surgery using CFS and GST_5m_, both previously used frailty assessment tools in clinical setting (Aucoin et al. 2020; Rockwood et al. 2005; Afilalo et al. 2010; Turner and Clegg 2014; Wilson et al. 2013). CFS was categorised as ‘Non-frail’ (CFS ≤ 4) and ‘Frail’ (CFS > 4) (Rockwood et al. 2005). For GST_5m_, patients were asked to walk 5 m at a comfortable pace and the walking time recorded (Afilalo et al. 2010). This test was repeated 3 times and the mean time calculated. High-risk status for frailty and poor outcome was defined as taking 6 seconds (s) or more to complete the 5-m distance (Afilalo et al. 2010).

The postoperative recovery outcome measured was days (alive and) at home within 30 days of surgery (DAH_30_), a patient-centred composite measure that incorporates the postoperative hospital length of stay, discharge destination (rehabilitation centre or nursing home), hospital readmission, and postoperative death (Myles et al. 2017; Moonesinghe et al. 2019). Construct validity has been established in perioperative studies involving cardiac surgical patients with half a day difference in DAH_30_ considered clinically meaningful (Myles et al. 2017).

Demographic data included age, gender, height, weight, haemoglobin and albumin levels, physical performance (including lower limb strength using the 30-s chair rise test (Rikli and Jones 1999), and total weekly physical activity level using the International Physical Activity Questionnaire (Macfarlane et al. 2007)), predicted mortality using the logistic European System for Cardiac Operative Risk Evaluation (EuroScore) (Roques et al. 2003), details of the surgical procedure, duration of anaesthesia, cardiopulmonary bypass time, ICU admission severity of illness (Acute Physiology and Chronic Health Evaluation III (Knaus et al. 1991)), duration of mechanical ventilation postoperatively, major adverse cardiac and cerebrovascular events, ICU and hospital length of stay, and 30-day mortality.

### Statistical analyses

Based on the preliminary results from the ongoing PREQUEL trial (Yau et al. 2019), 10% of study patients were expected to be frail (i.e. CFS > 4). A sample size of 85 patients provided 80% power to determine whether a correlation coefficient (0.30) between muscle ultrasound findings and frailty differs from zero with a 2-sided α error of 0.050.

Descriptive statistics with mean (SD), or median (IQR) for continuous variables, and count (percentage) for categorical variables were reported. The Shapiro–Wilk test was used to check data for normality. Comparisons of ultrasound measurements between frailty groups were examined using Student’s t-test or Mann–Whitney U test as appropriate. The Chi-squared test was used to compare categorical data between CFS groups. To test the reliability of ultrasound measurements, the intraclass correlation coefficient (ICC) was used to test interrater reliability between the study operator and the experienced radiologist. Repeated ultrasound assessments were performed on five patients on two separate occasions by the two operators during the same day. Spearman’s rho correlation (*r*_*s*_) and Pearson correlation (*r*) were estimated between CFS and GST_5m_ respectively with ultrasound measurements to determine their relationship. The receiver-operating characteristic analysis was performed to determine and compare the discriminative ability of each ultrasound measurement variable (MT_RFM_, CSA_RFM_ and Echo_RFM_) to identify frailty using the criteria of CFS > 4 and GST_5m_ ≥ 6 s. Exploratory cut-offs for each ultrasound measurement variable were estimated using the Youden’s index and the corresponding performance measures: sensitivity, specificity, positive and negative likelihood ratios, and area under receiver-operating characteristic curve (AUROC) with associated 95% confidence intervals (95% CI) were reported. The receiver-operating characteristic analysis was also used to determine and compare the discriminative performance of the various frailty measures for the prediction of DAH_30_.

Finally, the predictive performance of each of the ultrasound RFM measurements in combination with GST_5m_ as an add-on test to another objective test (GST_5m_) for identifying frailty was assessed, and compared with the two index measures (CFS and GST_5m_). The ‘both test positive’ rule was used to evaluate if the add-on tests (i.e. combining two objective assessment tools: GST_5m_ ≥ 6 s followed by the ultrasound-derived RFM measures at the threshold for frailty) increased the specificity (Hayen et al. 2010). For the purposes of this study, CFS was considered to be the reference test for frailty as it is extensively used to provide predictive screening for clinical outcomes, including in cardiac surgery and ICU settings (Shimura et al. 2017; Afilalo et al. 2017; Muscedere et al. 2017). McNemar’s test was used to compare the difference in diagnostic yield (proportion of true-positives in the study population), sensitivity (%), and specificity (%) between each add-on test and GST_5m_ alone. The relative positive and negative likelihood ratios were calculated to determine if the add-on tests outperformed the GST_5m_ alone test (Hayen et al. 2010). The performance of CFS, GST_5m_, and add-on test to predict DAH_30_ was also estimated. Using quantile regression with robust standard errors (Staffa et al. 2019), the changes in DAH_30_ distribution from 10 to 90th percentiles between CFS, GST_5m_ and the best add-on test frailty measure across frailty groups were described after adjusting for age, sex and logistic EuroScore. Calibration belts (Nattino et al. 2014) were drawn to assess the calibration performance of the four Firth logistic regression models of frailty measures (CFS or best add-on test) on DAH_30_ (binary outcome cut-off at 10th percentile or 50th percentile), adjusting for age, sex and logistic EuroScore. Statistical analyses were performed using SPSS software version 26 (IBM, New York), Stata software version 17 (StataCorp, College Station) and MedCalc software version 20.023 (MedCalc Software, Ostend). The level of significance was set at *p* < 0.050.

## Results

Between April 2020 and May 2021, 109 patients were screened. Ninety-six eligible patients consented to participate of which 85 completed the preoperative ultrasound examination and other assessments (Fig. 2). Seventy-nine patients were followed up to 30 days after surgery.

Eleven (13%) and 29 (34%) participants were classified as frail before surgery using CFS > 4 and GST_5m_ ≥ 6 s criteria respectively. Of the 74 non-frail patients (CFS ≤ 4), 42 (56.8%) were pre-frail (CFS = 4). Perioperative patient characteristics are shown in Table 1. Non-frail participants (CFS ≤ 4) had better lower limb strength (*p* = 0.008) and higher weekly physical activity level (*p* = 0.008) than frail participants (CFS > 4) preoperatively. Median (IQR) albumin concentration was similar between non-frail (38 g/dl [36–40]) and frail groups (38 g/dl [36–40]) before surgery (*p* = 0.848). All patients were able to walk independently.

**Table 1:** Perioperative characteristics of 85 participants by Clinical Frailty Scale

**Preoperative characteristics**	**Total** **(*n* = 85)**	**Non-frail** (CFS ≤ 4) **(*n* = 74)**	**Frail** (CFS > 4) **(*n* = 11)**	***p* value**
Mean (SD) age; *y*	64.2 (7.6)	64.2 (7.4)	64.0 (9.1)	0.922
Sex; male; n (%)	62 (73%)	56 (76%)	6 (55%)	0.159
Mean (SD) BMI; *kg.m*^*−2*^	25.4 (3.8)	25.7 (3.7)	23.2 (4.2)	0.039
Mean (SD) BSA; *m*^*2*^	1.75 (0.20)	1.77 (0.20)	1.63 (0.16)	0.028
Occupation; n (%)				0.117
- Working	25 (29%)	23 (31%)	2 (18%)	
- Housewife	18 (21%)	14 (19%)	4 (36%)	
- Unemployed	10 (12%)	7 (9%)	3 (27%)	
- Retired	32 (38%)	30 (41%)	2 (18%)	
Education level; n (%)				0.262
- Primary or below	35 (41%)	28 (38%)	7 (64%)	
- Secondary	47 (55%)	43 (58%)	4 (36%)	
- University or above	3 (4%)	3 (4%)	0 (0%)	
Home living status; n (%)				1.000
- Lives alone	3 (4%)	3 (4%)	0 (0%)	
- Lives with others	82 (96%)	71 (96%)	11 (100%)	
Median (IQR) stands within 30 s in CRT	9 (8–12)	10 (8–12)	7 (6–10)	0.008
Median (IQR) total weekly activity level; *METs.hour*^*−1*^ *.week*^*−1*^	23.1 (9.9–46.2)	23.8 (11.6–51.1)	11.6 (1.7–19.8)	0.008
Median (IQR) Hb level; *g.dl*^*−1*^	13.3 (12.4–14.4)	13.4 (12.7–14.5)	12.2 (11.7–14.2)	0.114
**Postoperative characteristics**	**Total** ***(n* = 79)**	**Non-frail** (CFS ≤ 4) ***(n* = 69)**	**Frail** (CFS > 4) ***(n* = 10)**	***p*** **value**
Type of surgery; n (%)				0.146
—CABG	36 (46%)	33 (48%)	3 (30%)	
—Valve	33 (42%)	27 (39%)	6 (60%)	
—CABG + Valve	10 (13%)	9 (13%)	1 (10%)	
Median (IQR) logistic EuroScore; *%*	3.1 (1.5–5.3)	2.7 (1.5–4.9)	4.4 (2.9–6.7)	0.084
Median (IQR) duration of surgery; *min*	256 (229–302)	275 (231–309)	237 (194–270)	0.066
Mean (SD) duration of anaesthesia; *min*	309 (62)	313 (64)	280 (42)	0.114
Median (IQR) duration of CPB; *min*	115 (95–138)	115 (96–143)	111 (92–142)	0.685
Mean (SD) APACHE III score	48.9 (11.5)	48.1 (10.8)	54.1 (15.2)	0.125
Median (IQR) duration of mechanical ventilation; *min*	505 (340–754)	495 (335–728)	638 (341–1075)	0.288
Median (IQR) length of stay in ICU; *hours*	21.7 (19.7–23.7)	21.6 (18.9–23.4)	23.5 (21.5–27.5)	0.047
Major cardiac and cerebrovascular events; n (%)	5 (6%)	3 (4%)	2 (20%)	0.118
Median (IQR) duration of hospital stay; *days*	11 (9–14)	10 (9–14)	16 (11–20)	0.026
Median (IQR) DAH_30_; *days*	21 (17–23)	22 (18–24)	14 (5–21)	0.007

*APACHE* Acute Physiology and Chronic Health Evaluation, *BMI* Body mass index, *BSA* Body surface area, *CABG* Coronary artery bypass grafting, *CFS* Clinical Frailty Scale, *CPB* Cardiopulmonary bypass, *CRT* Chair rise test, *DAH*_*30*_ Day (alive and) at home within 30 days of surgery, *Hb* Haemoglobin, *ICU* Intensive care unit, *MET* Metabolic equivalents, *min* Minute

Good-to-excellent interrater reliability between the study operator and radiologist for MT_RFM_ (ICC 0.85, 95% CI 0.60–0.95) and CSA_RFM_ (ICC 0.85, 95% CI 0.46–0.95) was found. There was excellent intra-rater reliability for measurements of MT_RFM_ (ICC [95% CI]: 0.99 [0.98–0.99]; 0.95 [0.92–0.96], respectively), CSA_RFM_ (ICC [95% CI]: 1.00 [1.00–1.00]; 1.00 [1.00–1.00], respectively), and Echo_RFM_ (ICC [95% CI]: 0.87 [0.81–0.91]; 0.85 [0.79–0.89], respectively) of the dominant and non-dominant legs.

**Fig. 4:**
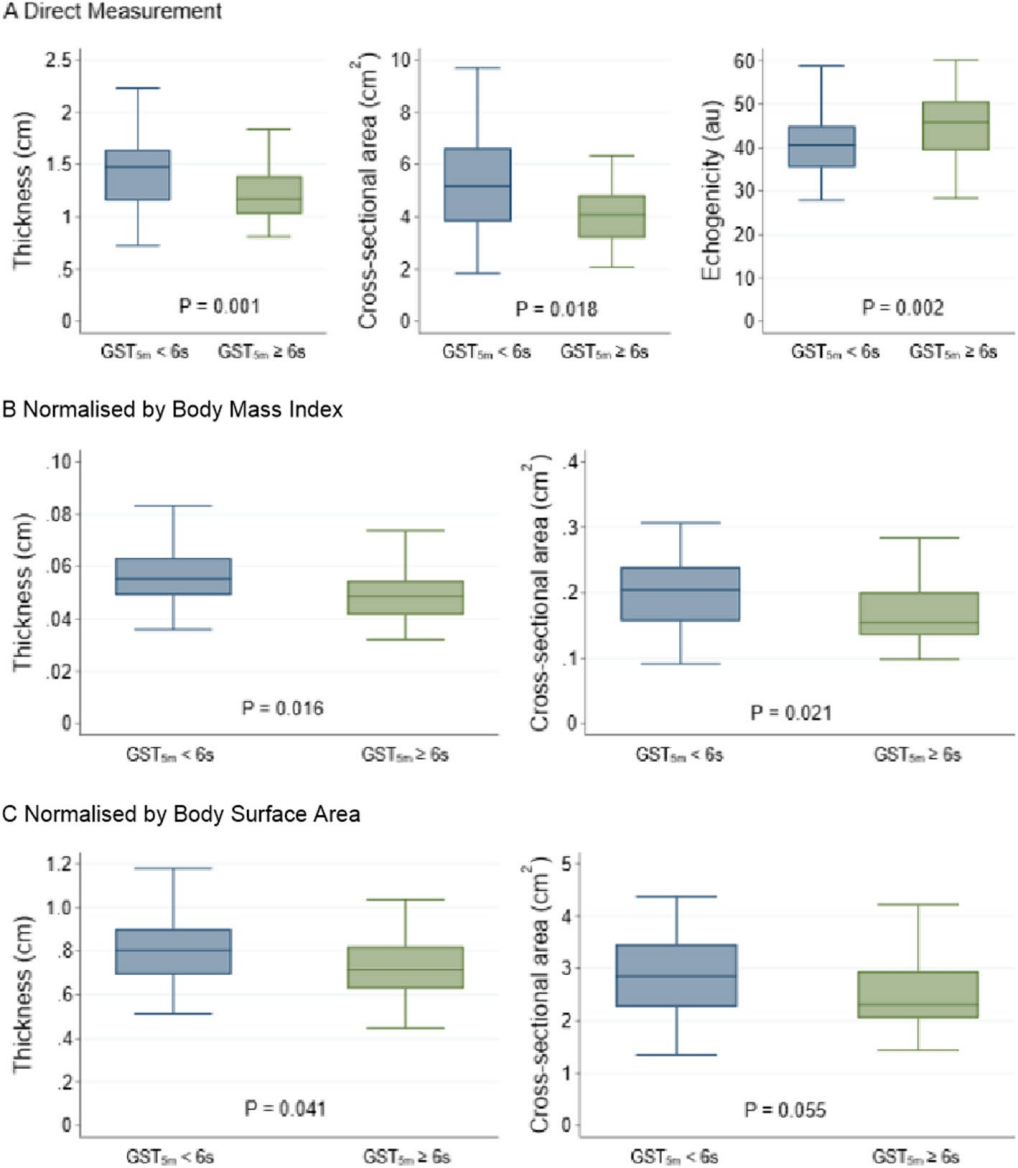
Box-and-whisker plots showing differences in ultrasound measurements between frail (GST_5m_ ≥ 6 s) and non-frail (GST_5m_ < 6 s) participants. **A** Actual values from direct measurement **B** normalised values by body mass index **C** normalised values by body surface area

The relationship between MT_RFM_, CSA_RFM_ and Echo_RFM_ measurements and the pre-defined frailty criteria of CFS and GST_5m_ are shown in Figs. 3 and 4 respectively. The mean MT_RFM_ and CSA_RFM_ of frail participants was significantly lower than those of non-frail participants (Figs. 3 and 4). There was weak correlation between frailty defined by CFS and all RFM measures: MT_RFM_ (*r*_*s*_ = -0.25, 95% CI -0.44 to -0.04), CSA_RFM_ (*r*_*s*_ = -0.26, 95% CI -0.45 to -0.05), and Echo_RFM_ (*r*_*s*_ = 0.19, 95% CI -0.03 to 0.39). There was moderate correlation between frailty defined by GST_5m_ and MT_RFM_ (*r* = -0.36, 95% CI -0.53 to -0.16), and GST_5m_ and CSA_RFM_ (*r* = -0.35, 95% CI -0.53 to -0.15), but weak correlation between GST_5m_ and Echo_RFM_ (*r* = 0.22, 95% CI 0.01 to 0.42).

There was acceptable discriminative performance for MT_RFM_ (AUROC 0.75, 95% CI: 0.64 to 0.84) and CSA_RFM_ (AUROC 0.76, 95% CI: 0.66 to 0.85) for frailty (CFS > 4), with a marginally worse performance if normalised by body mass index and body surface area (Table 2). The optimal cut-off for discriminating frail from non-frail patients for MT_RFM_ and CSA_RFM_ was ≤ 1.40 cm and ≤ 3.015 cm^2^ respectively. The discriminative performance for Echo_RFM_ was poor (AUROC 0.61, 95% CI: 0.50–0.72; Table 2) and had an optimal cut-off at > 45.85 au. The discriminative performance of MT_RFM_, CSA_RFM_ and Echo_RFM_ for GST_5m_ are shown in Supplementary Table S1.

**Table 2:** Receiver-operating characteristic analysis and cut-off thresholds for RFM ultrasound measurements for predicting frailty (CFS > 4)

	**Cut-off**	**Sensitivity** (95% CI)	**Specificity** (95% CI)	**Positive Likelihood Ratio** (95% CI)	**Negative Likelihood Ratio** (95% CI)	**AUROC** (95% CI)
**Muscle thickness**; *cm*						
Mean of dominant and non-dominant legs	≤ 1.400	90.9 (58.7–99.8)	50.0 (38.1–61.9)	1.82 (1.35–2.44)	0.18 (0.03–1.19)	0.75 (0.64–0.84)
Mean (normalised by BMI)	≤ 0.046	54.6 (23.4–83.3)	78.4 (67.3–87.1)	2.52 (1.26–5.04)	0.58 (0.30–1.12)	0.64 (0.53–0.74)
Mean (normalised by BSA)	≤ 0.880	100.0 (71.5–100.0)	28.4 (18.5–40.1)	1.40 (1.21–1.61)	0.00	0.66 (0.55–0.76)
**Muscle cross-sectional area**; *cm*^*2*^						
Mean of dominant and non-dominant legs	≤ 3.015	63.6 (30.8–89.1)	93.2 (84.9–97.8)	9.42 (3.62–24.53)	0.39 (0.18–0.85)	0.76 (0.66–0.85)
Mean (normalised by BMI)	≤ 0.190	90.9 (58.7–99.8)	52.7 (40.7–64.4)	1.92 (1.42–2.61)	0.17 (0.03–1.13)	0.74 (0.63–0.83)
Mean (normalised by BSA)	≤ 2.056	63.6 (30.8–89.1)	87.8 (78.2–94.3)	5.23 (2.45–11.17)	0.41 (0.19–0.91)	0.75 (0.64–0.83)
**Muscle echogenicity**; *au*						
Mean of dominant and non-dominant legs	> 45.85	54.6 (23.4–83.3)	75.7 (64.3–84.9)	2.24 (1.14–4.39)	0.60 (0.31–1.16)	0.61 (0.50–0.72)

*Au* Arbitrary units, *AUROC* Area under receiver-operating characteristic curve, *BMI* Body mass index, *BSA* Body surface area, *CFS* Clinical Frailty Scale, *RFM* Rectus femoris muscle

When two objective assessment tools were combined in a stepwise manner (GST_5m_ positive followed by RFM ultrasound examination positive), the GST_5m_ + CSA_RFM_ add-on test (Table 3) demonstrated the best positive likelihood ratio of 40.36 (95% CI: 5.25 to 304.29), but at the expense of a worse negative likelihood ratio of 0.46 (95% CI: 0.24 to 0.88) compared to the GST_5m_ alone test. Relative positive and negative likelihood ratios for GST_5m_ + MT_RFM_ and GST_5m_ + Echo_RFM_ add-on tests are shown in Supplementary Tables S2 and S3 respectively; all results were nonsignificant suggesting that add-on tests provided no additional gain in diagnostic test performance than single index test.

**Table 3:** Performance characteristics of GST_5m_ and add-on test (GST_5m_ ≥ 6 s followed by CSA_RFM_ ≤ 3.015 cm^2^) to identify frailty (CFS > 4)

				**Comparison between add-on test and** **GST**_**5m**_** test alone**	
	**GST**_**5m**_** test alone**	**CSA**_**RFM**_** test alone**	**Add-on test**	**Difference**	***p*** value
**Yield**					
No./total	9/85	7/85	6/85		
% (95% CI)	10.6 (5.0 to 19.2)	8.2 (3.4 to 16.2)	7.1 (2.6 to 14.7)	-3.5 (-13.5 to 6.3)	0.419
**Sensitivity**					
No./total	9/11	7/11	6/11		
% (95% CI)	81.8 (52.3 to 94.9)	63.6 (30.8–89.1)	54.6 (28.0 to 78.73)	-27.3 (-60.8 to 16.4)	0.180
**Specificity**					
No./total	54/74	69/74	73/74		
% (95% CI)	73.0 (62.2 to 82.2)	93.2 (84.9–97.8)	98.7 (93.6 to 100.0)	25.7 (14.0 to 37.5)	< 0.001
**AUROC** (95% CI)	0.77 (0.65 to 0.90)	0.76 (0.66–0.85)	0.77 (0.62 to 0.92)	-0.01 (-0.15 to 0.14)	0.913
**Likelihood ratio**				**Relative LR**	
Positive (95% CI)	3.03 (1.90 to 4.83)	9.42 (3.62–24.53)	40.36 (5.25 to 304.29)	13.32 (1.67 to 105.94)	0.014
Negative (95% CI)	0.25 (0.07 to 0.88)	0.39 (0.18–0.85)	0.46 (0.24 to 0.88)	1.84 (0.44 to 7.63)	0.401

*AUROC* Area under receiver-operating characteristic curve, *CFS* Clinical Frailty Scale, *CSA*_*RFM*_ Muscle cross-sectional area of the rectus femoris muscle, *GST*_*5m*_ 5-m gait speed test, *LR* Likelihood ratio

Median (IQR) DAH_30_ was 21 days (17-23). Patients with CFS-defined frailty, spent less days at home than non-frail patients (*p* = 0.007) (Table 1). Patients with GST_5m_-defined frailty also had less days at home than non-frail patients (median [IQR] DAH_30_: 18 days [11-21] vs 22 days [19-24], *p* < 0.001). The discriminatory performance of CFS, GST_5m_, ultrasound-derived measures, and individual add-on tests for DAH_30_ is shown in Table 4. The GST_5m_ + CSA_RFM_ add-on test had the best diagnostic test performance (AUROC 0.90, 95% CI 0.81–0.95), with high specificity (94.4, 95% CI 86.4–98.5) and positive likelihood ratio of 12.86 (95% CI 4.45–37.10) and may be superior to either CFS or gait speed test alone (Table 4). Simultaneous quantile regression models of DAH_30_ on the best add-on test (GST_5m_ + CSA_RFM_) and on CFS, with corresponding Firth logistic regression discrimination (AUROC) and calibration belts performance examined at the 10th percentile (poor recovery) and 50th percentile DAH_30_ are shown in Supplementary Figure S1.

**Table 4:** Receiver-operating characteristic analysis of CFS, GST_5m_, ultrasound-derived measurements and add-on test for predicting DAH_30_

	**Sensitivity** (95% CI)	**Specificity** (95% CI)	**Positive Likelihood Ratio** (95% CI)	**Negative Likelihood Ratio** (95% CI)	**AUROC** (95% CI)
**Single frailty tests**					
CFS > 4	50.0 (18.7–81.3)	94.2 (85.8–98.4)	8.62 (2.77–26.84)	0.53 (0.28–0.99)	0.76 (0.66–0.85)
GST_5m_ ≥ 6 s	81.5 (61.9–93.7)	63.5 (49.0–76.4)	2.23 (1.49–3.33)	0.29 (0.13–0.66)	0.73 (0.62–0.83)
Muscle thickness (MT_RFM_) ≤ 1.40 cm	62.8 (46.7–77.0)	61.1 (43.5–76.9)	1.61 (1.01–2.58)	0.61 (0.38–0.97)	0.63 (0.51–0.73)
Muscle cross-sectional area (CSA_RFM_) ≤ 3.015 cm^2^	45.5 (16.7–76.6)	94.1 (85.6–98.4)	7.73 (2.45–24.4)	0.58 (0.34–1.00)	0.64 (0.52–0.74)
Muscle echogenicity (Echo_RFM_) > 45.85 au	53.6 (33.9–72.5)	78.4 (64.7–88.7)	2.48 (1.33–4.65)	0.59 (0.39–0.90)	0.66 (0.55–0.77)
**Add-on test**					
GST_5m_ + CSA_RFM_	71.4 (29.0–96.3)	94.4 (86.4–98.5)	12.86 (4.45–37.16)	0.30 (0.09–0.98)	0.90 (0.81–0.95)

*Au* Arbitrary units, *AUROC* Area under receiver-operating characteristic curve, *CFS* Clinical Frailty Scale, *CSA*_*RFM*_ Muscle cross-sectional area of the rectus femoris muscle, *DAH*_*30*_ Days (alive and) at home within 30 days of surgery, *Echo*_*RFM *_Muscle echogenicity of the rectus femoris muscle, *GST*_*5m*_ 5-m gait speed test, *MT*_*RFM*_ Muscle thickness of the rectus femoris muscle

## Discussion

The main finding of this study was that in adult patients awaiting cardiac surgery, ultrasound measurement of the RFM cross-sectional area is moderately related to frailty (defined as CFS > 4)_._ The novel stepwise, ‘add-on’ test (i.e. GST_5m_ plus RFM ultrasound examination) was more predictive of DAH_30_ than either the CFS, GST_5m_ or any of the single RFM ultrasound measures. Both univariate and multivariate analyses identified the associations between CFS and GST_5m_ + CSA_RFM_ add-on test and poor recovery, defined as DAH_30_ less than or equal to 11 days, with very good to excellent discrimination and satisfactory calibration performances. Although CFS is widely used in different settings, possibly because of its ease and efficiency for clinical or research use, the scoring of CFS is criticised for its subjective nature and dependence on patient recall. The use of the proposed add-on test strategy (i.e. GST_5m_ plus RFM ultrasound examination) offers an objective measure of frailty that can potentially identify frail patients and better predict DAH_30_, a meaningful patient-centred outcome.

This study was designed to be pragmatic, and therefore RFM ultrasound measurements were obtained by a front-line healthcare worker (a physiotherapist) who was instructed in hands-on ultrasound techniques to enable the acquisition of reliable measurements of the RFM. The training period was relatively short, comprising of three weekly practice sessions (each lasting 60 min) with five supervised patient scans performed over a period of about one month, however, the training could be reasonably completed in one week if required. The operator performance measurements indicated a high level of procedural accuracy was obtained. The relative ease of training, operator ability achieved and improved discriminatory performance of the add-on test combining gait speed and ultrasound findings for meaningful patient outcome suggest that the use of ultrasound examination of the RFM has potential for clinical use in high-risk cardiac patients awaiting surgery.

Other studies have investigated the ability of RFM ultrasound measurements to predict frailty and adverse outcome risk. In patients admitted to a surgical ICU, Mueller et al. (Mueller et al. 2016) found that ultrasound measurements of RFM cross-sectional area correlated well with frailty, and predicted a poor outcome. Two recent studies have investigated the role of lower limb muscle ultrasound measurements in identifying patients with frailty and those at high risk of surgical complications. Salim and colleagues (Salim et al. 2020) measured the thigh muscle thickness (normalised to thigh length) by ultrasound in a group of 49 elderly (> 64 years) patients undergoing abdominal surgery. They found an inverse correlation with CFS-defined frailty and major postoperative complications concluding that thigh ultrasound should be further tested as an objective tool to assess frailty (Salim et al. 2020). While their study was similar to the current study, notable differences were uncertainty as to who performed the ultrasound examination, and measurements were taken 3–5 days postoperatively rather than preoperatively (Salim et al. 2020). Lastly, as only correlations were explored, the potential clinical utility of the method was difficult to demonstrate. Interestingly, however, by making a direct comparison between ultrasound and computerised tomography measurements, they did show that ultrasound was comparable to computerised tomography for detecting muscle mass loss (Salim et al. 2020).

In a comparative study of 32 patients scheduled for major non-cardiac surgery and 20 healthy volunteers, Canales and colleagues (Canales et al. 2022) found that preoperative ultrasound measurements of quadriceps depth and RFM cross-sectional area were able to discriminate between frail and non-frail patients prior to surgery. These measurements had a moderate ability to predict delirium risk, length of ICU stay, and the need for unplanned admission to a high care facility (Canales et al. 2022). The findings were similar to those of the current study except that measurements were obtained by a board-certified ultrasonographer rather than a physiotherapist with limited, focused training over a one-month period. Making use of staff that are already part of the perioperative team to perform ultrasound examination involves minimal disruption to workflow and will limit the inconvenience and extra cost of utilising a board-certified ultrasound operator.

This study helps confirm the applicability of ultrasound-based leg muscle assessment to recognise patients at risk of frailty, and classify patients into high-risk groups for adverse outcomes. These risks were specifically confirmed in a preoperative cardiac population, showing how already engaged healthcare providers such as a physiotherapist, can be trained in a relatively short time to accurately measure key muscle parameters such as RFM thickness, cross-sectional area and echogenicity. Our findings, together with recently published work in perioperative general surgical patients suggest that ultrasound-based assessment of frailty may be an effective strategy for preoperative risk stratification.

The study has several limitations. First, despite being the largest observational study to date in the preoperative population, the prevalence of frailty based on the CFS criterion was relatively low. Second, the sample size was primarily designed for evaluating the relationship between ultrasound examination of the RFM and frailty measures, and postsurgical recovery and postoperative physical performance indicators were not assessed. Third, only one trained operator was utilised in our study, and therefore more data will be required to establish whether ultrasound studies performed by similarly trained operators will have consistently acceptable diagnostic and predictive performance, and satisfactory interrater/operator reliability. Lastly, while other authors normalised measurements utilising such adjustment factors as BMI and BSA (Canales et al. 2022; Salim et al. 2020), normalisation of our data did not substantially improve predictive performance.

## Conclusions

This prospective cohort study found that the preoperative ultrasound examination of the RFM, in particular cross-sectional area, was moderately associated with frailty in patients undergoing cardiac surgery, and was a good predictor for poor postoperative recovery outcome. The predictive performance was further improved when RFM measurements were combined with the use of an objective screening tool of muscle function, the GST_5m_.

### Supplementary Information

Supplementary Mateial 1.

## Data Availability

The datasets analysed during the current study are available in the CUHK Research Data Repository, 10.48668/PAXAKV/0EYHM9

## Abbreviations

95% CI: 95% Confidence intervals
AUROC: Area under receiver-operating characteristic curve
BMI: Body mass index
BSA: Body surface area
CFS: Clinical Frailty Scale
CSA_RFM_: Muscle cross-sectional area
DAH_30_: Days (alive and) at home within 30 days of surgery
Echo_RFM_: Muscle echogenicity
EuroScore: European System for Cardiac Operative Risk Evaluation
GST_5m_: 5-Metre gait speed test
ICC: Intraclass correlation coefficient
ICU: Intensive care unit
MT_RFM_: Muscle thickness
RFM: Rectus femoris muscle
s: Second
