# Supplementary material for: Preoperative rectus femoris muscle ultrasound, its relationship with frailty scores, and the ability to predict recovery after cardiac surgery: a prospective cohort study
Source: Perioper Med (Lond). 2024 May 23;13:45. doi: 10.1186/s13741-024-00401-y (PMC11112902; doi:10.1186/s13741-024-00401-y)
Supplement: Supplementary file 1 — Supplementary Mateial 1. [file 13741_2024_401_MOESM1_ESM.docx]

**SUPPLEMENTARY TABLES AND FIGURE**

Preoperative rectus femoris muscle ultrasound for identifying frailty and predicting recovery after cardiac surgery: a prospective cohort study

**Supplementary Table S1.** Receiver-operating characteristic analysis and cut-off thresholds for RFM ultrasound measurements for predicting frailty (GST_5m_≥6s)

|  | **Cut-off** | **Sensitivity**  (95% CI) | **Specificity**  (95% CI) | **Positive Likelihood Ratio**  (95% CI) | **Negative Likelihood Ratio**  (95% CI) | **AUROC**  (95% CI) | |
| --- | --- | --- | --- | --- | --- | --- | --- |
| **Muscle thickness**; cm | | | | | | |  |
| Mean of dominant and non-dominant legs | ≤1.420 | 86.2  (68.3-96.1) | 58.9  (45.0-71.9) | 2.10  (1.49-2.97) | 0.23  (0.09-0.60) | 0.71  (0.60-0.81) | |
| Mean (normalised by BMI) | ≤0.049 | 58.6  (38.9-76.5) | 75.0  (61.6-85.6) | 2.34  (1.36-4.05) | 0.55  (0.35-0.87) | 0.66  (0.55-0.76) | |
| Mean (normalised by BSA) | ≤0.785 | 72.4  (52.8-87.3) | 55.4  (41.5-68.7) | 1.62  (1.12-2.34) | 0.50  (0.26-0.94 | 0.64  (0.53-0.74) | |
| **Muscle cross-sectional area**; cm^2^ | | | | | | |  |
| Mean of dominant and non-dominant legs | ≤4.825 | 75.9  (56.5-89.7) | 57.1  (43.2-70.3) | 1.77  (1.23-2.55) | 0.42  (0.21-0.84) | 0.66  (0.55-0.76) | |
| Mean (normalised by BMI) | ≤0.202 | 79.3  (60.3-92.0) | 53.6  (39.7-67.0) | 1.71  (1.22-2.39) | 0.39  (0.18-0.82) | 0.65  (0.54-0.75) | |
| Mean (normalised by BSA) | ≤2.613 | 65.5  (45.7-82.1) | 58.9  (45.0-71.9) | 1.60  (1.06-2.40) | 0.59  (0.34-1.01) | 0.63  (0.52-0.73) | |
| **Muscle echogenicity**; au | | | | | | |  |
| Mean of dominant and non-dominant legs | >44.12 | 69.0  (49.2-84.7) | 73.2  (59.7-84.2) | 2.57  (1.57-4.23) | 0.42  (0.24-0.75) | 0.70  (0.59-0.80) | |

*au = arbitrary units; AUROC = area under receiver-operating characteristic curve; BMI = body mass index; BSA = body surface area; GST_5m_ =*

*5-metre gait speed test; RFM = rectus femoris muscle*

**Supplementary Table S2.** Performance characteristics of GST_5m_ and add-on test (GST_5m_≥6s followed by MT_RFM_≤1.40 cm) to identify frailty (CFS>4)

|  |  |  |  | **Comparison between add-on test and**  **GST_5m_ test alone** | | |
| --- | --- | --- | --- | --- | --- | --- |
|  | **GST_5m_ test alone** | **MT_RFM_ test alone** | **Add-on test** | **Difference** | **p value** | |
| **Yield** |  |  |  |  |  |  |
| No./total | 9/85 | 10/85 | 9/85 |  |  | |
| % (95% CI) | 10.6 (5.0 to 19.2) | 11.8 (5.8 to 20.6) | 10.6 (5.0 to 19.2) | 0 (-10.5 to 10.5) | 1.000 | |
| **Sensitivity** |  | | | | |  |
| No./total | 9/11 | 10/11 | 9/11 |  |  | |
| % (95% CI) | 81.8 (52.3 to 94.9) | 90.9 (58.7 to 99.8) | 81.8 (52.3 to 94.9) | 0 (-37.2 to 37.2) | 1.000 | |
| **Specificity** |  | | | | |  |
| No./total | 54/74 | 37/74 | 59/74 |  |  | |
| % (95% CI) | 73.0 (62.2 to 82.2) | 50.0 (38.1 to 61.9) | 79.7 (69.2 to 87.3) | 6.8 (-7.9 to 21.1) | 0.339 | |
| **AUROC** (95% CI) | 0.77 (0.65 to 0.90) | 0.75 (0.64 to 0.84) | 0.81 (0.69 to 0.93) | 0.03 (0.00 to 0.06) | 0.023 | |
| **Likelihood ratio** |  |  |  | **Relative LR** |  | |
| Positive (95% CI) | 3.03 (1.90 to 4.83) | 1.82 (1.35 to 2.44) | 4.04 (2.37 to 6.86) | 1.33 (0.66 to 2.70) | 0.425 | |
| Negative (95% CI) | 0.25 (0.07 to 0.88) | 0.18 (0.03 to 1.19) | 0.23 (0.06 to 0.80) | 0.92 (0.15 to 5.63) | 0.928 | |

*AUROC = area under receiver-operating characteristic curve; CFS = Clinical Frailty Scale; GST_5m_ = 5-metre gait speed test; LR = likelihood ratio; MT_RFM_ = muscle thickness of the rectus femoris muscle*

**Supplementary Table S3.** Performance characteristics of GST_5m_ and add-on test (GST_5m_≥6s followed by Echo_RFM_>45.85 au) to identify frailty (CFS>4)

|  |  |  |  | **Comparison between add-on test and**  **GST_5m_ test alone** | |
| --- | --- | --- | --- | --- | --- |
|  | **GST_5m_ test alone** | **Echo_RFM_ test alone** | **Add-on test** | **Difference** | **p value** |
| **Yield** |  |  |  |  |  |
| No./total | 9/85 | 7/85 | 6/85 |  |  |
| % (95% CI) | 10.6 (5.0 to 19.2) | 8.2 (3.4 to 16.2) | 7.1 (2.6 to 14.7) | -3.5 (-13.5 to 6.3) | 0.419 |
| **Sensitivity** |  |  |  |  |  |
| No./total | 9/11 | 6/11 | 5/11 |  |  |
| % (95% CI) | 81.8 (52.3 to 94.9) | 54.6 (23.4-83.3) | 45.5 (21.3 to 72.0) | -36.4 (-67.5 to 9.0) | 0.083 |
| **Specificity** |  |  |  |  |  |
| No./total | 54/74 | 56/74 | 65/74 |  |  |
| % (95% CI) | 73.0 (62.2 to 82.2) | 75.7 (64.3-84.9) | 87.8 (78.5 to 93.5) | 14.9 (1.0 to 28.1) | 0.024 |
| **AUROC** (95% CI) | 0.77 (0.65 to 0.90) | 0.61 (0.50 to 0.72) | 0.67 (0.51 to 0.82) | -0.11 (-0.26 to 0.04) | 0.161 |
| **Likelihood ratio** |  |  |  | **Relative LR** |  |
| Positive (95% CI) | 3.03 (1.90 to 4.83) | 2.24 (1.14-4.39) | 3.74 (1.53 to 9.11) | 1.23 (0.45 to 3.38) | 0.682 |
| Negative (95% CI) | 0.25 (0.07 to 0.88) | 0.60 (0.31-1.16) | 0.62 (0.36 to 1.07) | 2.48 (0.63 to 9.84) | 0.196 |

*au = arbitrary units; AUROC = area under receiver-operating characteristic curve; CFS = Clinical Frailty Scale; Echo_RFM_ = muscle echogenicity of the rectus femoris muscle; GST_5m_ = 5-metre gait speed test; LR = likelihood ratio*


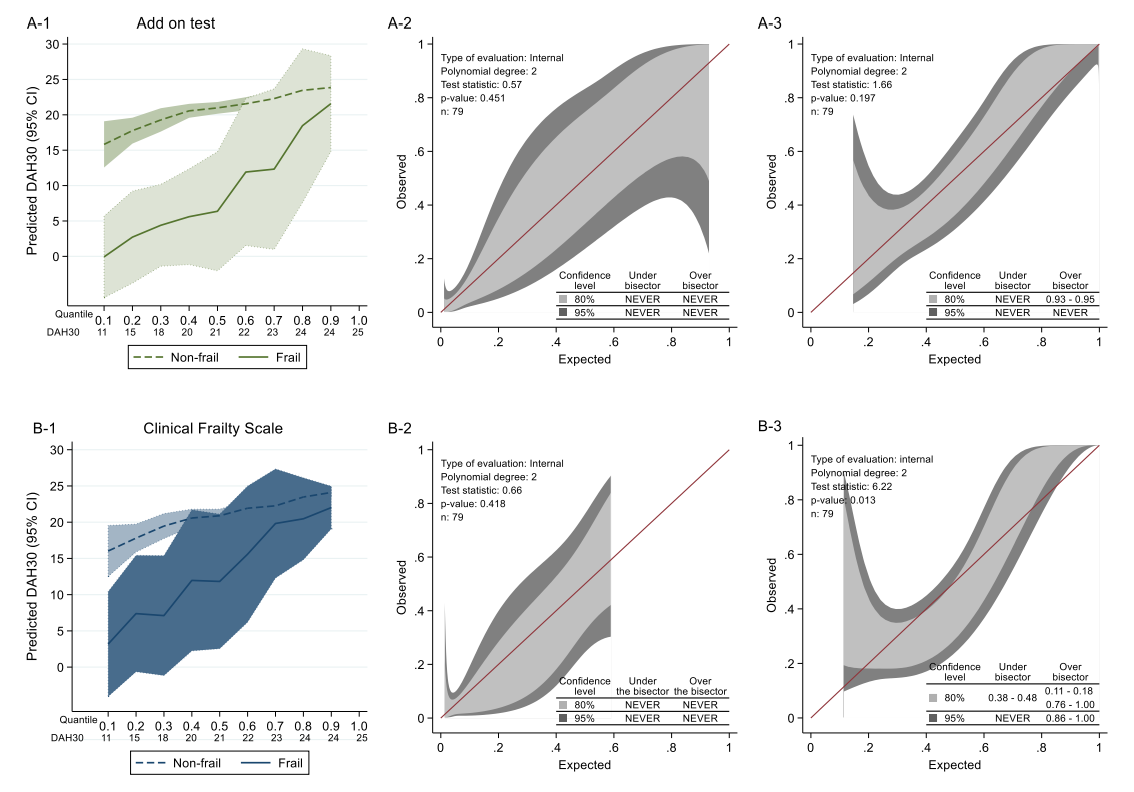
**Supplementary Figure S1.** Simultaneous quantile regression models of DAH_30_ on the best add-on test (GST_5m_ + CSA_RFM_) and CFS

(A-1) Simultaneous quantile regressions of days (alive and) at home within 30 days after surgery (DAH_30_) on add-on test with 5-metre gait speed test (GST_5m_) and ultrasound-derived cross-sectional area of the rectus femoris muscle (CSA_RFM_) (*X-axis shows quantile levels from 0.10 to 0.90 with corresponding observed DAH_30_*); (A-2) Calibration belts after performing Firth logistic regression on add-on test (GST_5m_ + CSA_RFM_) at DAH_30_ cut-off at 11 days (*AUROC 0.91, 95% CI: 0.78- 0.97*) and (A-3) at 21 days (*AUROC 0.78, 95% CI: 0.66-0.86*) adjusting for age, sex and logistic EuroScore.

(B-1) Simultaneous quantile regressions of DAH_30_ on Clinical Frailty Scale (CFS) (*X-axis shows quantile levels from 0.10 to 0.90 with corresponding observed DAH_30_*); (B-2) Calibration belts after performing Firth logistic regression on CFS at DAH_30_ cut-off at 11 days (*AUROC 0.83, 95% CI: 0.62-0.97*) and (B-3) at 21 days (*AUROC 0.79, 95% CI: 0.70-0.90*) adjusting for age, sex and logistic EuroScore.
